# Identification of Targets to Redirect CAR T Cells in Glioblastoma and Colorectal Cancer: An Arduous Venture

**DOI:** 10.3389/fimmu.2020.565631

**Published:** 2020-09-30

**Authors:** Eleonora Ponterio, Ruggero De Maria, Tobias Longin Haas

**Affiliations:** ^1^Fondazione Policlinico Universitario “A. Gemelli” —Istituti di Ricovero e Cura a Carattere Scientifico, Rome, Italy; ^2^Istituto di Patologia Generale, Università Cattolica del Sacro Cuore Rome, Rome, Italy; ^3^IIGM—Italian Institute for Genomic Medicine, IRCCS, Candiolo, Italy; ^4^Candiolo Cancer Institute, Fondazione del Piemonte per l'Oncologia-Istituti di Ricovero e Cura a Carattere Scientifico, Candiolo, Italy

**Keywords:** solid tumor, CRC (colorectal cancer), CAR T cells therapy, CSCs, GBM, MAbs

## Abstract

The chimeric antigen receptor (CAR) is an artificial molecule engineered to induce cytolytic T cell reactions in tumors. Generally, this molecule combines an extracellular single-chain variable fragment (scFv) able to recognize tumor-associated epitopes together with the intracellular signaling domains that are required for T cell activation. When expressed by T cells, the CAR enables the recognition and subsequent destruction of cancer cells expressing the complementary antigen on their surface. Although the clinical application for CAR T cells is currently limited to some hematological malignancies, researchers are trying to develop CAR T cell-based therapies for the treatment of solid tumors. However, while in the case of CD19, or other targets restricted to the hematopoietic compartment, the toxicity is limited and manageable, the scarcity of specific antigens expressed by solid tumors and not by healthy cells from vital organs makes the clinical development of CAR T cells in this context particularly challenging. Here we summarize relevant research and clinical trials conducted to redirect CAR T cells to surface antigens in solid tumors and cancer stem cells with a focus on colorectal cancer and glioblastoma. Finally, we will discuss current knowledge of altered glycosylation of CSCs and cancer cells and how these novel epitopes may help to target CAR T cell-based immunotherapy in the future.

## Introduction

The three traditional pillars of cancer treatment, surgery, radiotherapy and chemotherapy are still the therapy of choice for most patients (1). The immunotherapy treatments approved in recent years has widened the arsenal to the fight against cancer (2, 3), particularly for the use of monoclonal antibodies (mAbs) and genetically modified cells recognizing tumor-associated antigens (TAAs) (4). In some cases, immunotherapy results in significant improvement of the patient survival, even when the disease was particularly resistant to the traditional therapies (5, 6). Among the different cellular immunotherapy strategies, the adoptive transfer of T cells directed against tumor antigens is a new and particularly promising approach for the rapid generation of many tumor-specific lymphocytes (7). The transduction of T cells with a chimeric antigen receptor (CAR) recognizing TAAs is an effective method to target tumor cells in an MHC-independent manner. The clinical outcome of the CAR T cell approach in solid tumors depends on several parameters (8) such as CAR architecture (9); lymphodepletion before the administration of CAR T cells (10); efficient tumor homing and persistence in the tumor environment (11, 12); toxicity (13); specificity for the target (14). Most of these parameters are extensively reviewed in the cited articles. In this review we will give first a brief overview about the molecular composition of the CARs and then concentrate on the tumor targeting and the lack of specific antigens as one of the biggest difficulties in the generation of CAR T cell therapy in general and particularly in solid tumors such as colorectal cancer (CRC) and glioblastoma (GB) (7).

## The Molecular Composition of Chimeric Antigen Receptor-An Overview

Under physiological conditions, the specificity of T cells is strictly dictated by the recognition of major histocompatibility complex (MHC)-presented antigen by the T cell receptor (TCR) and subsequent clonal expansion of antigen-specific (e.g., tumor-specific) cells. Using recombinant DNA technologies and retro- or lentiviral transduction, T lymphocytes can be engineered to express CARs. These consist of an extracellular domain that serves for antigen recognition and an intracellular domain for signal transduction. In the majority of CARs, the central component used for the signal transduction is derived from the CD3 zeta chain (CD3z) of the TCR complex, while the antigen recognition is directed by a single-chain variable fragment (scFv) engineered from antibody heavy and light chains (9). These structures combine the specificity of MHC-independent antibody recognition with the anti-tumor potential of T lymphocytes and open the possibility to generate T lymphocytes of any antigenic specificity. CARs using only the CD3z chain for signal transduction are defined as first-generation (15, 16). T lymphocytes expressing these constructs show strong anti-tumor activity *in vitro*, but they have limited efficacy *in vivo* (17). These observations led to the design of second-generation CARs, which are engineered with an additional intracellular costimulatory domain often derived from either CD28, 4.1BB, ICOS, or OX40 molecules. The transduction with second-generation CARs produces T cells that have a greater capacity for cytokine production and expansion (18, 19). The combination of three signal domains (e.g., CD3z-CD28-4.1BB or CD3z-CD28-OX40) further increased the activity. These constructs are subsequently called third-generation CARs (20–22). The so-called fourth-generation CARs or TRUCKs (CAR T cells redirected for universal cytokine killing) have shown to increase T cell activation, proliferation, and persistence, through the combination of two costimulatory domains and the engineered capability of enhanced cytokine secretion (23, 24). However, although third and fourth generation CARs were shown to have advantages in preclinical model systems, their superiority compared to second-generation CARs in the clinical setting still has to be proven. We also like to mention that the only two FDA approved CAR therapies, tisagenlecleucel (KYMERIAH) and axicabtagene ciloleucel (YESCART) are both based on second-generation constructs. In addition to the classification by how the activating signal is transduced, the CAR can be differentiated based on its capacity to recognize a single or several TAAs. To increase the versatility, universal CARs (UniCARs) and tandem CARs (tanCARs) were developed. UniCARs have an extracellular moiety that binds to a soluble adaptor, which in turn defines the specificity against a certain TAA. Several different versions of UniCARs with adaptable specificity are available. These include antibody-dependent cytotoxicity receptors such as NKp30 (targeting B7H6) (25), CD16 (26), and NKG2D (27). The anti-Tag CARs also belong to the UniCARs. These receptors utilize scFvs targeting molecular tags or chemically conjugated peptides, which in turn bind to tumor antigens (28) and are supplied either systemically or intratumoral in the experimental animal. A similar strategy is followed by the biotin-binding immune receptor CAR (BBIR CAR) that employs the biotin-avidin system to bind CAR T cells to an antigen (29) In these constructs, the extracellular scFv part is replaced by a biotin-binding protein (e.g., avidin). This allows for the simultaneous targeting of multiple antigens by exogenous addition of different biotinylated ligands recognizing TAAs (e.g., antibodies). BBIR CAR T cells have been shown to result in tumor suppression, both *in vitro* and *in vivo* (29, 30). The split, universal, and programmable (SUPRA) CARs follow a similar strategy by linking the antigen-binding molecule (scFv) with the help of a leucine-zipper oligomerization system to the transmembrane and intracellular activation domain of the CAR. This system was shown to be very versatile as several ligands can be employed (31). However, although the versatility of the UniCARs is intriguing, their transfer into the clinical setting may be impaired by several caveats. For the generation of SUPRA CARs, the transduction of several expression cassettes is needed. This may lead to substantial technical problems in the generation and standardization of the cells. Furthermore, the potential immunogenicity of the leucine zippers is likely to be higher as of standard scFv-CARs. This problem of the increased immunogenicity and thus neutralization may also affect the BBIR CARs that consist of a non-human, potentially highly immunogenic biotin-binding domain and the tags needed by the ligands for the anti-Tag CARs (32). TanCARs can be used to overcome these problems. TanCARs induce distinct T cell reactivity against two different tumor-restricted antigens and result in a synergistic enhancement of effector functions when both antigens are simultaneously encountered (33–35). A major advantage of this system is that the tandem CAR preserves the cytolytic ability of T cells even upon loss of one of the target molecules and thus, reduces the risk of antigen escape that is a substantial problem for CAR T cell therapy.

By the time of this review, clinical benefits of CAR T cell treatments have mainly been observed in B cell malignancies such as relapsed B cell acute lymphoblastic leukemia (B-ALL) and diffuse large B cell lymphoma (DLBCL) (36, 37). Apart from the comparable easy accessibility of the tumor cells, the nature of the antigens that serve as targets for the CARs has strongly contributed to the therapy success. Most CARs generated for these tumors target the CD19, CD20, and CD22 (35), that are highly expressed on the tumor cells and thus enable a potent on-target/on-tumor effect of the CAR T cells. However, these molecules are also present during B cell development and the most evident on-target/off-tumor effect of the treatment results in B cell depletion. Fortunately, this effect can be managed by immunoglobulin replacement, and the clinical benefit of the massive anti-tumor function justifies the risks of side effects (38).

## CAR T Cells Targeting Tumor-Associated Antigens in Colorectal Cancer and Glioblastoma

The identification of suitable surface antigens in solid tumors is more complicated and currently under heavy investigation (39). Of over 671 ongoing clinical trials in the CAR T field, the U.S. National Library of Medicine (ClinicalTrials.gov) database listed 189 CAR T cell trials targeting solid tumors at the time of this review. To reduce the complexity, we will here concentrate on CAR T cell targets explored in clinical trials of two important solid tumor entities: GB that represents the most aggressive form of brain tumors and CRC, which is the third most deadly tumor type worldwide (40, 41). Tables 1, 2 give an overview of ongoing CAR T cell trials for CRC and GB that are currently recruiting patients in the United States, Europe and China. Here we will introduce the CAR T cells and their targets that are currently being investigated in these clinical trials.

**Table 1:** Selected CAR T cell clinical trials for CRC.

**Target**	**Identifier**	**Tumor**	**Country**	**N**	**Results**
NKR2	NCT03018405	CRC	USA/Europe	146	Recruiting, not disclosed
	NCT03310008	mCRC	Europe/Belgium	36	Active, non-recruiting
	NCT03370198	mCRC	Europe/Belgium	1	Active, non-recruiting
NKG2D	NCT03692429	mCRC	Europe/Belgium	36	Recruiting, not-disclosed
CD133	NCT02541370	CRC	China	20	Completed, not-disclosed
CEA	NCT02349724	CRC	China	75	Unknown, not-disclosed
	NCT03682744	CRC	United States	18	Active, not recruiting
EGFR	NCT01869166, NCT03152435	CRC	China	60	Unknown, not disclosed
EGFRvIII	NCT03267173	CRC	China	10	Unknown, not disclosed
EpCAM	NCT03013712	CRC	China	60	Recruiting, not disclosed
MUC1	NCT02617134	CRC	China	20	Unknown, not disclosed

*Natural-killer 2 Receptor (NKR2); Natural-killer group 2, member D receptor protein (NKG2D); CD133; Carcinoembryonic antigen (CEA); Epidermal growth factor receptor (EGFR); Epidermal growth factor receptor variant III (EGFRvIII); Epithelial cellular adhesion molecule (EpCAM); Mucin 1 (MUC1)*.

**Table 2:** Selected CAR T cell clinical trials for GB.

**Target**	**Identifier**	**Tumor**	**Country**	**N**	**Results**
NKG2D	NCT04270461	GB	USA	10	Not yet recruiting
CD147	NCT04045847	GB	China	31	Not recruiting
B7H3	NCT04077866	GB	China	40	Not yet recruiting
EGFRVIII	NCT02844062	GB	China	20	Unknown, not disclosed
	NCT02664363	GB	United States	3	Terminated, not disclosed
	NCT03726515	GB	United States	7	Active, not recruiting
	NCT01454596	GB	United States	18	Completed, results (closed)
EpHA2	NCT02575261	GB	China	60	Completed, not disclosed
GD2	NCT03252171	GB	China	60	Completed, not disclosed
HER2	NCT01109095	GB	United States	16	Completed, not disclosed
	NCT03389230	GB	United States	42	Recruiting, not disclosed
IL13Rα2	NCT04003649	GB	United States	60	Recruiting, not disclosed
	NCT02208362	GB	United States	92	Recruiting, not disclosed

*B7H3; CD147; Epidermal growth factor receptor variant III (EGFRvIII); EPH receptor A2 (EpHA2); Disialoganglioside 2 (GD2); HER2; Interleukin 13 receptor α2 (IL13Rα2), Natural-killer group 2, member D receptor protein (NKG2D)*.

### CAR T Cells Targeting CRC

The surface protein ERBB2, epidermal growth factor receptor 2 (HER2) is a member of the tyrosine kinase receptors family and is highly expressed by many cancer cells (42). NC03740256 is a phase 1 trial in combination with an oncolytic adenovirus (CAdVEC). CAdVEC supports the immune system including HER2-specific CAR T cells to react against the tumor by promoting a pro-inflammatory microenvironment. Another member of the family of tyrosine kinase receptors, epidermal growth factor receptor (EGFR) also appears to be a good target for CRC (43) and also GB (see below). Recently two clinical trials were launched to evaluate the targeting of this protein in phase I and phase II (NCT03152435 and NCT01869166 CRC). However, by the time of this review, no results of these studies were available.

Several clinical trials are investigating, the use of carcinoembryonic antigen (CEA) CAR T cells in different tumors including CRC. Zhang et al. demonstrated the safety and efficacy of a CAR T cell therapy targeting CEA-positive CRC patients with lung and liver metastases in a phase I trial. They demonstrated that CEA CAR T cell therapy was well tolerated in CEA+ CRC patients even in high doses, and some efficacy was observed in most of the treated patients (44).

In this dose-escalation trial seven out of 10 patients initially showed stable disease by PET or CT analyses. In two of them, the tumor growth was inhibited for more than 30 weeks (44).

In another clinical trial, the feasibility of delivering first-generation CAR T cell therapy to patients with advanced CEACAM5+ malignancy was determined (NCT01212887). Unfortunately, no objective clinical responses were observed. Instead, the on-target/off-tumor toxicity against pneumocytes and lung-associated macrophages was so high that the trial had to be closed (17).

The epithelial cell adhesion molecule (EpCAM) is aberrantly expressed in several epithelial-derived tumors including CRC (45, 46) and also suggested as a target for CAR T or NK cells. In preclinical studies, an EpCAM second-generation CAR was constructed and transduced into NK-92 cells by lentiviral vectors. Synergistic effects of regorafenib and EpCAM CAR NK-92 cells were analyzed in a mouse model with human colorectal cancer xenografts. The CAR NK-92 cells specifically recognized EpCAM-positive colorectal cancer cells, released cytokines including IFN-γ, perforin, and granzyme B, and showed cytotoxic activity *in vitro* (47). These results encouraged the launch of a clinical trial with CAR T cells recognizing EpCAM positive cells in CRC as well as hematological malignancies (NCT03013712). This trial was designed as phase I/II and is still ongoing.

Hedge et al. reported a clinical trial with patients with metastatic CRC who have been treated in two phase I trials with first-generation retroviral transduced CAR T cells targeting tumor-associated glycoprotein (TAG)-72. Both trials (C-9701 and C9702) were not successful, and the limited persistence of the cells was supported by the finding that the tumor-associated TAG-72 expression is non-uniform. Unfortunately, the data from these CART72 trials did not give any insight some insight into whether coadministration of IFN-α can result in sufficient TAG-72 upregulation to avoid the loss of antigen (48).

Finally, a Mucin-1 (MUC1) CAR T cell therapy was proposed for metastatic colorectal adenocarcinoma. It was shown to be safe in humans (49) and is now investigated in a phase I/II trial (NCT02617134) with over 73 participants. This trial consists of multi-target-gene-modified CAR/TCR T cells.

### CAR T Cells Targeting GB

At the time of this review, several CAR T cell trials targeting different proteins in GB are ongoing (Table 2). By now, published results of these trials are only available for some of the targets.

A robust anti-tumor efficacy following regional intraventricular delivery of HER2-CAR T cells for the treatment of multifocal brain metastases and leptomeningeal disease was described (50). The HER2-CAR T cells persisted for 6 weeks without evident toxicities. Although this therapy was designed to target breast cancer metastases, the data demonstrated the safety and feasibility of intraventricular HER2 CAR T cell administration and showed encouraging signals of clinical activity (51), thus setting the stage for studies that combine HER2-CAR T cells with other immune-modulatory approaches to enhance their expansion and persistence (51, 52). The re-stimulation of antiviral immunity via defined peptides from common pathogens provides a unique therapeutic avenue for cancer immunotherapy. Reactivating the virus-specific memory T cells (VSTs) arrested the growth of checkpoint blockade-resistant and poorly immunogenic tumors in mice after injecting adjuvant-free non-replicating viral peptides into tumors (53). These results extend recent observations of virus-specific T cells in GB. In a clinical study of 17 patients with progressive HER2-positive GB, autologous HER2-specific CAR-modified VSTs were infused without prior lymphodepletion (NCT01109095). The treatment with VSTs was safe and well-tolerated, with no dose-limiting toxic effects. Seven patients showed stable disease after CAR T cell treatment and three showed long term responses of more than 2 years without progression (54). While these studies are very encouraging for intracranial applications, the systemic treatment with high-affinity HER2-CARs can also be dangerous. A patient with metastatic colon cancer received an infusion of CAR T cells targeted to the antigen HER2 (ERBB2) and died 5 days later (55) due to the massive on-target/off-tumor toxicity of the CAR T cells for lung cells that express low levels of HER2. Moreover, in an animal model, similar problems were also observed for CAR T cells with high affinity for Disialoganglioside 2 (GD2, glycolipid antigen) (56) which has been identified as an immunotherapy target in melanoma and neuroblastoma about 10 years ago (57, 58). Although this antigen serves as a bona fide model that the affinity of the targeting may be tightly associated with unwanted toxicity, the treatment with lower affinity CAR T cells showed much promise in recent studies in diffuse midline gliomas (DMGs) with mutated histone H3 K27M (H3-K27M). If the results can be translated into humans, it could be a valuable immunotherapeutic strategy for children with H3-K27M-mutant DMGs (59).

Another intensively studied GB associated tumor antigen is interleukin 13 receptor α2 (IL13Rα2) (60) which was described as a potential CAR target more than 10 years ago (61). Subsequent studies showed efficacy in animal models (62, 63). Interestingly, one of these studies showed a higher expression of IL13Rα2 on stem-like vs. differentiated glioma populations, indicating that IL13Rα2-directed immunotherapeutic approaches could be useful for eradicating therapeutically resistant glioblastoma stem cell (GSC) populations (62). IL13Rα2 was the primary target in two important clinical studies showing safety and efficacy in humans (60, 64). In contrast to most CARs, of which some also target IL13Rα2 (65), the binding domain of the construct used in these studies was not an scFv. but based on IL13 fused to the intracellular signaling domains. Thus, these CARs also recognize interleukin receptor 13 alpha 1 (IL13Rα1), and this dual specificity most probably resulted in a strong therapeutic effect. In the study of Brown et al. the described patient was a participant in an ongoing dose-escalation safety study to evaluate the role of intracranial CAR T cell therapy targeting IL13Rα2 in patients with malignant gliomas. However, after stunning initial responses, the tumor relapsed most likely due to the antigen loss (60, 66).

Epidermal growth factor receptor deletion mutant variant III (EGFRvIII) is a tumor-specific antigen expressed in GB and its expression is often associated with survival, invasion, angiogenesis and resistance to radio- and chemotherapy (67). Sampson et al. developed a third-generation, EGFRvIII-specific murine CAR, and performed tests to determine its efficacy in a fully immunocompetent mouse model of malignant glioma. They showed that CAR-treated, cured mice were resistant to rechallenge with EGFRvIII negative tumors, suggesting the generation of host immunity against additional tumor antigens (68). These results in a refined syngeneic mouse model suggested that EGFRvIII-targeted CAR T cells may provide a highly specific, promising therapeutic candidate for patients with tumors in the CNS and a phase I clinical trial (NCT01454596) was launched. Unfortunately, this study with 10 patients failed. Two of the patients treated with the highest doses of CAR T cells experienced severe hypoxia and one of these patients died. No objective responses were detected nor persistent CAR+ cells were identified (69). While the molecular reason for the toxicity remains unclear, an explanation for the lack of clinical efficacy may be the heterogeneity of antigen expression (70, 71) and a different activation of bystander immune cells in mouse and human. Thus, antigen loss would be one of the main reasons for the lack of therapeutic efficacy observed in the clinical trials. Furthermore, studies by Maus and colleagues showed that a single dose of peripherally infused CAR T cells targeting EGFRvIII resulted in marked antigen loss and reduced clinical efficacy of this treatment (72).

In summary, the clinical results obtained for CAR T cell therapy in CRC and GB are much less promising when compared to B cell leukemia. The studies indicate that besides other T cell-intrinsic or environmental factors (73–77), the risks of on-target/off-tumor toxicity and antigen loss are two of the main problems hindering a long-lasting therapeutic success. Thus, strategies to optimize CAR T cell function in solid tumors including the discovery of new targets remain an important goal and will be discussed in the sections below.

## Strategies to Improve the Targeting of CARs to CRC and GB

**Figure 1:**
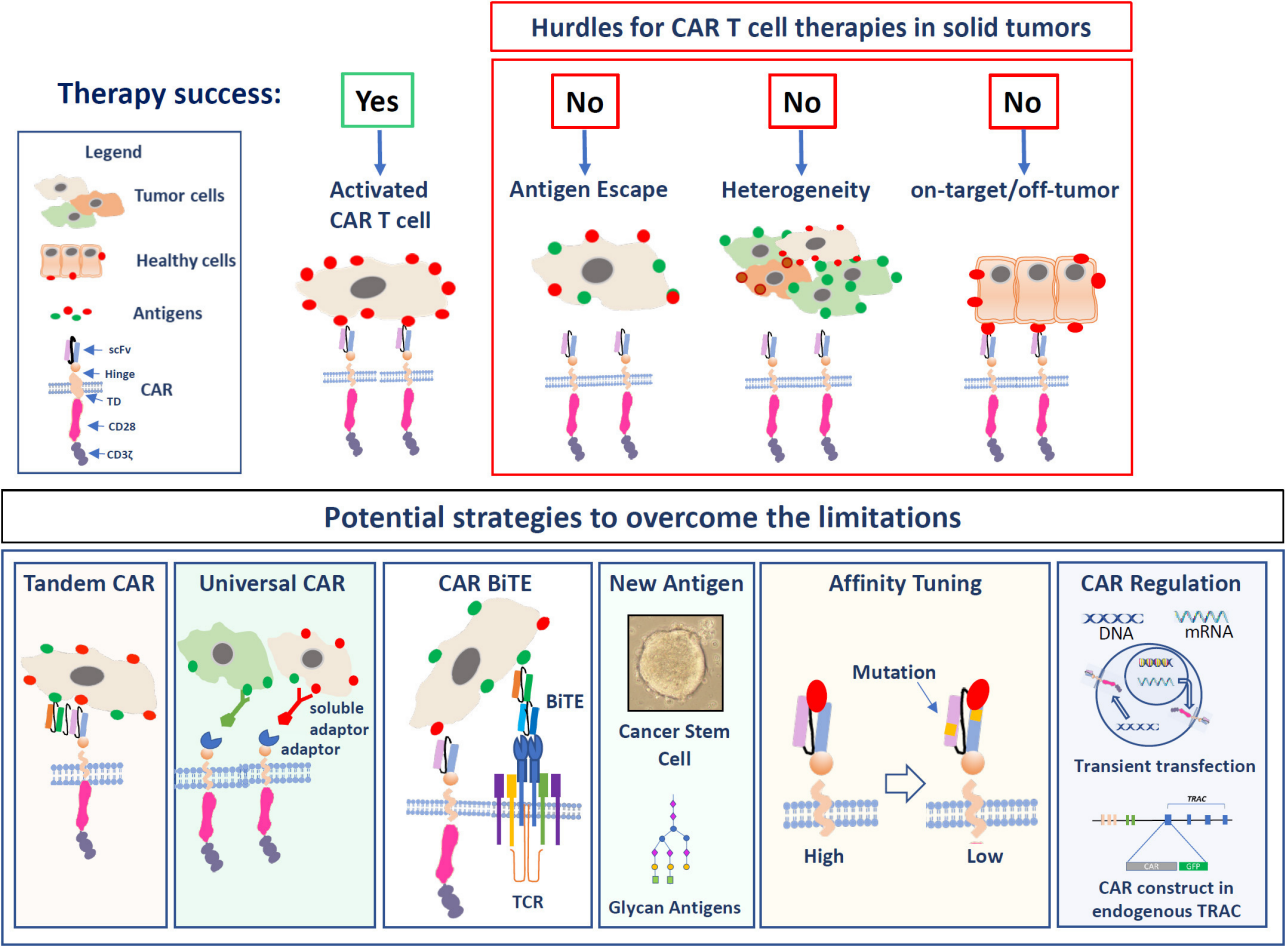
The figure illustrates the hurdles of solid tumor targeting as well as potential strategies to overcome these limitations. Upper panel: The major hurdles are: Antigen escape, tumor cells that lose the expression of the antigen; Heterogeneity due to the expression of different TAAs on solid cancers; On-target/off-tumor toxicity in the case that antigens are also highly expressed in healthy cells. In the lower panel, the strategies to optimize CAR T cell function in solid tumors are illustrated: Targeting the T cells with Tandem CARs, universal CARs, or BiTEs. Targeting alternative antigens, Affinity tuning, and the regulation of the CAR expression levels.

A precise tumor targeting and the lack of specific antigens is one of the biggest difficulties in the generation of CAR T cell therapy in general and particularly in solid tumors (7). The absence of cancer-specific targets increases the potential risk of significant on-target/off-tumor toxicity in case that antigens are also expressed in healthy tissues (78). These problems and some of the potential solutions are summarized in Figure 1. A potential solution for this problem can be the use of CARs modified to bind antigens highly expressed by tumor cells and present, although at lower levels, in healthy tissues with lower affinity. By introducing mutations in the antigen-binding regions of the scFv, Liu et al. generated CARs binding to HER2 and EGFR with lower affinity. The resulting CAR T cells still killed the TAA overexpressing tumor cells efficiently but are likely to be much less toxic for healthy cells. A similar result was shown by two EGFR-targeting CARs generated with the scFvs from two monoclonal antibodies of different affinities for the antigen (79, 80). These results together with the route of application (e.g., intracranial vs. systemic) may also explain why some HER2-targeting therapies are well-tolerated, while others showed fatal side effects (54, 55). A strong impact of the affinity on potential toxicity was also observed with GD2-CAR T cells. Richman et al. showed that inserting a single amino acid exchange in the scFv (E101K) was generating GD2-CARs with 10-fold more affinity to the target. Compared to the CARs with the lower affinity scFv, these cells were much more efficient in killing GD2-expressing cells *in vitro*. Unfortunately, the treatment with these high-affinity CAR T cells resulted in fatal encephalitis in the mouse model, while the CARs generated with the wt scFv were not toxic (56). Other ways to enhance the safety of CARs recognizing TAAs also expressed by healthy tissues is a stringent control of their expression levels either by transient expression methods using mRNA transfer instead of stable transduction (81) or the integration of the transgene in a defined genetic locus enabling controlled expression levels using the CRISPR/Cas9 technology (82).

Another problem is that the few known highly tumor-specific TAAs are often lost during the treatment, which reduces their therapeutic value. To overcome some of these problems, the concept of utilizing bi-specific tanCARs is very attractive for fighting solid tumors. The ideal antigen should be selected based on high cell surface expression in cancerous tissue and low cell surface expression on healthy tissue. As an example, HER2, MUC1, and EpCAM are not highly expressed by normal colon tissues and their co-expression should be limited to cancerous tissue. Using this approach, T cells were transduced with both a CAR that provided suboptimal activation upon binding of one antigen and a chimeric costimulatory receptor (CCR) that recognized a second antigen (83). Although this is a very interesting concept, CCRs are so far still in the preclinical stage. To increase the specificity of CARs targeting GB, two or even three antigens were used. In one study, the antigen escape observed upon treatment with IL13-CARs was approached by the construction of second-generation tanCARs targeting IL13Rα2 and human epidermal growth factor receptor 2 (HER2) (66). These tanCAR T cells recognized tumors distinctly and effectively and improved persistence in the presence of both antigens (84). The single universal (U) tricistronic transgene (UCAR) T cells are generated by the expression of three independent CAR constructs in one T cell. Constructs for contemporary targeting of IL13Rα2, HER2, and EphA2 have shown some preclinical functions in mouse models (85). However, although this approach may be useful to overcome the antigen heterogeneity in GB and other tumors, it is not very likely that these treatments can be translated into the clinical setting. The transgenes are very large and complicate the generation of high titer virus and the integration of the viral genome needed for the genetic modification of the primary T cells under current clinical settings.

The problem of antigen escape can also be addressed by other CAR approaches. These include the induced expression of bi-specific T-cell engagers (BiTEs) or the use of UniCARs. BiTEs typically consist of two scFvs, one specific to CD3 (T cell co-receptor) and the other one specific to a tumor antigen, connected by a flexible linker. Thus, these molecules can physically link a T cell to a tumor cell (86). Choi and colleagues recently showed that BiTEs can enhance CAR T cell efficacy *in vivo*. They found a clearance of heterogeneous EGFRvIII/EGFR expressing GB cells in mouse models, by using a bicistronic construct to drive expression of a CAR specific for EGFRvIII, and a BiTE against wild type EGFR (74). The secreted EGFR-specific BiTEs were able to re-direct CAR T cells and recruited non-transduced bystander T cells against wild-type EGFR (74). Thus, BiTE-secreting CAR T cells hold much promise for the treatment of solid tumors and can provide an advantage over CAR T cells (28). As another strategy to improve the versatility and the safety of CAR T cell therapies, several groups used a CAR platform termed UniCAR system consisting of two components: UniCAR-modified T cells and tumor-specific target modules (TM). The bivalent α-EGFR-EGFR TM has shown to redirect UniCAR T cells to tumor cells expressing low levels of EGFR. According to PET experiments *in vivo*, the increased avidity of the bivalent α-EGFR-EGFR TM improves the enrichment at the tumor site (87).

While these approaches can help to increase the efficacy of CAR T cell therapy against known antigens, the identification of more robust targets with high potential to help the eradication of the tumor is still a major task in the fight against solid tumors.

## Search for Novel Antigens: Targeting of CAR T Cells to Cancer Stem Cells (CSCs) in Solid Tumors

The heterogeneity and thus high risk of antigen escape in solid tumors belong to the main cavets in the design of efficient CAR T cell therapies. A potential solution may be the selective targeting of tumor cell subpopulations that drive tumor growth. For GB and CRC, it is generally accepted that tumor growth is fueled by a subpopulation of CSCs that promote tumor progression and are highly resistant to conventional therapy (88). Thus, the extinction of these cells by CAR T cells represents a promising anti-tumor therapy. Interestingly, the primary cultures enriched in CSCs may be responsible that these cells keep many features of the primary tumor, including some tumor antigens (89). In the last 15 years, it was shown that CSCs from different solid tumors express various surface proteins at levels substantially higher when compared to the healthy or bulk tumor cell population (90). While all these markers may represent potential targets, by today only a limited number of CARs recognizing GB- and CRC-CSC surface markers are under investigation and will be discussed in this section.

Our group discovered that CRC metastases arise from disseminated colorectal cancer stem cells (CR-CSCs). Todaro et al. showed that CR-CSCs express CD44 variant 6 (CD44v6), which is required for their migration and generation of metastatic tumors (91).

CD44v6-CAR T cells have been generated to target leukemia and myeloma cells. These CAR T cells display potent *in vitro* and *in vivo* anti-tumor reactivity (92–94). However, because CD44v6 is also highly expressed in some normal tissues, especially in the skin, the safety of this treatment has to be proven before applying this therapy to humans.

EPH Receptor A2 (EphA2) is a tyrosine kinase (95) capable of activating multiple diverse signaling pathways involved in tissue homeostasis and cancer (96) and described as being a functional CSC marker in GB (97). CAR T cells targeting EphA2 showed a dose-dependent cell killing of esophageal squamous cell carcinoma (ESCC) cells and have been optimized for the adoptive T cell therapy of EphA2+ glioblastoma for further clinical development (98). Based on these results a clinical trial with EphA2-CAR T cells in GB was launched (NCT02575261).

In a preclinical study, the effect of NKG2D-CAR T cells on GB and GB stem cells was investigated and confirmed the high expression of NKG2DLs in all the samples. The NKG2D-BBz CAR T cells efficiently lysed GB cells and CSCs *in vitro* and produced high levels of cytokines, perforin, and granzyme B. The CAR T cells markedly eliminated xenograft tumors *in vivo* and did not exhibit significant treatment-related toxicity in the treated mice. In conclusion, NKG2D-CAR T cells targeted GB cells and CSCs, support the use of CAR T therapy in GB (99) and let to the design of a clinical trial (NCT04270461).

CD133 is a marker expressed by CSCs of various origins, including GB and CRC, and another attractive therapeutic target for cancers. The potential danger of CD133-CARs was unveiled in a study of Bueno et al. The authors treated mice with B-ALL and detected strong myeloablative toxicity upon CD133-CAR T cell transfer. Most probably this was due to the high expression of CD133 on the mouse hematopoietic stem cells (100). This toxicity was not detected in a phase I clinical trial (NCT02541370). Wang et al. showed the feasibility, controllable toxicities, and effective activity of CD133-CAR T transfer for treating patients with CD133-positive and late-stage metastatic malignancies. In this trial, 14 of 23 patients showed stable disease upon treatment and 3 even partial remissions. As described above, different affinities of the CARs targeting the murine and human protein may be responsible for the different toxicities (101).

While the concept of killing selectively the cells responsible for tumor growth and dissemination is very appealing, the targeting of CSCs by CAR T cells is being complicated by several factors. The vast majority of CSC-markers are also expressed on the surface of tissue-specific stem cells (102). This raises the likelihood of strong and potentially non-controllable on-target/off-tumor effects. A second problem may be that the low percentage of CSCs is surrounded by the tumor bulk and thus not accessible for the CAR T cells. Furthermore, there is accumulating evidence that CSCs can shift between stem and differentiated states depending on cell-intrinsic or microenvironmental factors. This “CSC plasticity” is also reflected by the expression of the stemness markers (103) that may be lost although the cells contain the capacity to self-renew and drive tumor growth. Thus, additional alternative antigens selectively expressed by the majority of the tumor cells need to be identified.

## Search for Novel Antigens: Targeting of Altered Glycan Structures in Cancer Cells

Many tumor- or CSC-selective monoclonal antibodies (mAbs) directly bind to the sugar chains of glycolipids (e.g., SSEA-3/4, GD2) or glycosylation-residues of proteins (e.g., some CD133 mAbs, CA 19-9). Compared to healthy tissue cancer cells have an altered metabolism leading to different repertoires of metabolites and activities of enzymes catalyzing glycosylation. This ultimately results in aberrant glycosylation patterns on their cell surface and secreted glycoproteins (104). Thus, these structures may represent a class of potential CAR antigens that, by now, received little attention. Glycans have fundamental mechanisms in controlling cancer development and progression (105). Changes in the cellular glycosylation are associated with malignant transformation of cancer cells, tumor progression and metastasis formation (106). Furthermore, glycans have a major impact on the interplay between cancer cells and the tumor microenvironment (104, 106, 107).

Compared to healthy tissue, CSCs and cancer cells have increased levels of truncated O-glycans (T and Tn antigens) and fucosylation, increased Lewis antigen expression and increased sialylation. All these altered structures could be new targets for CARs based on specific mAbs (48, 108–117). Some interesting mAbs targeting truncated O-glycan structures Tn and sialyl-Tn are characterized by relatively high affinity (~10–9 M range) and little or no reactivity against the peptide with elongated O-glycans or the non-glycosylated peptide (118) (Table 3).

**Table 3:** Selection of mAbs targeting glycosylation-related tumor-associated epitopes discovered in the last 20 years.

**MAB**	**CLASS**	**ANTIGEN**	**REFERENCE**
LpMab-21	O-Glycopeptide	Sialyl-PDPN	(119)
PankoMAb	O-Glycopeptide	MUC1	(120)
2D9	O-Glycopeptide	Tn-MUC1	(111)
6E3	O-Glycopeptide	Tn-MUC4	(121)
5E5	O-Glycopeptide	Tn-MUC1	(122)
mAb237	O-Glycopeptide	Tn-OTS8	(110)
5G2	N-Glycan	Le^a^ Le^c^	(115)

Schietinger et al. found that a wild-type transmembrane protein can be transformed into a TAA by a change of the glycosylation pattern. A somatic mutation in the chaperone gene Cosmc abolished the function of a glycosyltransferase, disrupted O-glycan Core 1 synthesis, created a tumor-specific neo-epitope consisting of a monosaccharide and the wild-type protein sequence. This epitope induced a high-affinity, highly specific, syngeneic mAb with anti-tumor activity (110). Sato et al. generated antibodies by directly immunizing mice with spheroids from human CRC. They obtained a functional mAb recognizing glycan structures that were lost in conventional cell lines. These results show that cancer tissue-originated spheroids can be a useful antigen for generating novel anti-cancer antibodies (123).

MUC1 is a large O-glycan-carrying protein over-expressed by most adenocarcinomas (124). MUC1-CAR T cells have been engineered based on the mAb 5E5 and have shown efficacy in eliminating pancreatic cancer cells (111). In a paper by Posey et al., the authors demonstrated the therapeutic efficacy of CAR T cells directed against Tn-MUC1 and presented aberrantly glycosylated antigens as a novel class of targets for tumor therapy with engineered T cells (114).

CD171 is an abundant cell surface molecule on neuroblastomas and a glycosylation-dependent tumor-specific epitope is recognized by the CE7 mAb. CE7-CAR T cell therapy was successful in 4 out of 5 neuroblastoma patients in a phase I study. All four CE7-CAR T cell products demonstrated *in vitro* and *in vivo* anti-tumor activity (117).

CAR T cells targeting stage-specific embryonic antigen 4 (SSEA-4) were also generated (125). The overexpression of SSEA-4 in several cancers including GB, the relatively restricted expression in normal tissues and anti-tumor effects of the antibody in preclinical mouse models in the absence of toxic side effects made it an interesting target. Unfortunately, the CAR T cell treatment in mice resulted in strong on-target/off-tumor effects especially in the hematopoietic stem cell pool (126).

Liau et al. produced an IgM antibody that is capable to distinguish malignant ovarian carcinoma cells from benign ovarian epithelia by binding specifically to cancer cell-associated glycans (127). Kaneho et al. developed and characterized anti-glycopeptide mAbs against human podoplanin hPDPN that is expressed in cancer cells or cancer-associated fibroblasts indicating poor prognosis (128).

Finally, disialoganglioside 2 (GD2, glycolipid antigen) (129, 130) has been identified as an immunotherapy target in melanoma and neuroblastoma about 10 years ago (57, 58). As reported above, this antigen serves as a bona fide model that the affinity of the targeting is tightly associated with unwanted toxicity (56). However, the treatment with lower affinity CAR T cells showed much promise in recent studies in diffuse midline gliomas (DMGs). Currently, a clinical trial targeting GD2 in GB was is recruiting patients (NCT03252171). In preclinical approach, a CAR targeting GD2 was also used to direct tumor necrosis factor-related apoptosis-inducing ligand (TRAIL) expressing mesenchymal stem cells into experimental GB. Although the results have still to be confirmed in more relevant systems, this approach shows potential new venues on how to fight GB with CARs (131).

## Conclusion and Future Directions

In recent years, CAR T cell immunotherapy has achieved encouraging results in the treatment of onco-hematological pathologies. Despite significant progress, some important challenges have not yet been resolved in treating solid tumors, especially in terms of specificity, persistence, safety and immunosuppressive microenvironment. In particular, the lack of tumor-selective antigens hinders the development of an efficient CAR T therapy for solid tumors. Although the expression of tumor-specific antigens is likely to be patient-specific and thus reliable biomarkers are needed to guide the therapy decisions, we assume that the identification of novel targets is one of the main keys to improve CAR T cell therapy for solid tumors such as GB and CRC. Besides modern gene expression-based approaches, we suggest applying primary tumor cultures enriched in CSCs to generate and screen for highly specific mAbs as for the engineering of novel CARs. We are convinced that CARs with mAbs targeting altered structures of cancer cells and CSCs offer a valid opportunity to develop new therapeutic options. Although significant barriers remain and hider the broad clinical application of CAR T in solid tumors, numerous studies are underway and more specific and safer CAR T cells can be expected in the future.

## Author Contributions

EP designed the review. EP and TLH wrote the main manuscript. RDM and TLH critically revised the manuscript. All authors have approved the final version of the manuscript and agree to be accountable for all aspects of the work.

## Conflict of Interest

The authors declare that the research was conducted in the absence of any commercial or financial relationships that could be construed as a potential conflict of interest.
